# Multi-omic integration with human dorsal root ganglia proteomics highlights TNFα signalling as a relevant sexually dimorphic pathway

**DOI:** 10.1097/j.pain.0000000000003656

**Published:** 2025-05-20

**Authors:** Allison M. Barry, Julia R. Sondermann, Joseph B. Lesnak, Feng Xian, Úrzula Franco-Enzástiga, Jayden A. O'Brien, David Gomez-Varela, Morgan K. Schackmuth, Stephanie Shiers, Theodore J. Price, Manuela Schmidt

**Affiliations:** a Department of Neuroscience and Center for Advanced Pain Studies, University of Texas at Dallas, Richardson, TX, United States; b Systems Biology of Pain, Division of Pharmacology & Toxicology, Department of Pharmaceutical Sciences, University of Vienna, Vienna, Austria

**Keywords:** Proteomics, Sexual dimorphism, Multi-omics, Human DRG

## Abstract

The peripheral nervous system (PNS) plays a critical role in pathological conditions, including chronic pain disorders, that manifest differently in men and women. To investigate this sexual dimorphism at the molecular level, we integrated quantitative proteomic profiling of human dorsal root ganglia (hDRG) and peripheral nerve tissue into the expanding omics framework of the PNS. Using data-independent acquisition (DIA) mass spectrometry, we characterized a comprehensive proteomic profile, validating tissue-specific differences between the hDRG and peripheral nerve. Through multi-omic analyses and in vitro functional assays, we identified sex-specific molecular differences, with TNFα signalling emerging as a key sexually dimorphic pathway with higher prominence in men. Genetic evidence from genome-wide association studies further supports the functional relevance of TNFα signalling in the periphery, while clinical trial data and meta-analyses indicate a sex-dependent response to TNFα inhibitors. Collectively, these findings underscore a functionally sexual dimorphism in the PNS, with direct implications for sensory and pain-related clinical translation.

## Abstract 2

Supplemental Digital Content is Available in the Text.

Using quantitative proteomics and cross-omic analyses of human dorsal root ganglia, we show evidence for sexual dimorphism in TNFα signalling spanning the epigenetic signature to proteomic differences.

## 1. Introduction

Neuroimmune- and pain-related condition prevalence, as well as the corresponding treatment efficacy, can differ across sex and gender.^27,40^ Understanding sexual dimorphism at a molecular level is thus a fundamental clinical issue. Autoimmune conditions, for example, are highly biased toward women (upwards of 80%), and infections can elicit differing immune responses. Women also have heightened sensitivity to pain in an experimental context, show higher rates of chronic pain, and are the dominant gender in many pain-centric disorders, from migraine to complex regional pain syndrome (CRPS).^8,15,40^

Preclinical work in rodents shows a similar sexual dimorphic trend at the immune and nervous system levels. Decades of work suggest complex, cross-species mechanism(s) underlying these clinical presentations. Hormones such as oestrogen, prolactin, and testosterone have been implicated in a range of pain and neuroimmune conditions, while evidence for central and peripheral sexually dimorphic mechanisms has been shown.^31,44,50,51,61^

Primary afferents in the peripheral nervous system (PNS) project from the skin and viscera to the dorsal horn of the spinal cord, with cell bodies in the dorsal root ganglia (DRG, in humans—hDRG). The DRG contain sensory neuron cell bodies, as well as a diverse set of non-neuronal cells. These sensory neurons respond to inflammation by detecting immune mediators and then releasing neuropeptides in complex neuroimmune circuits.^32,57^ At a molecular level, transcriptional profiles of these cell types have recently been published for human sensory neurons,^9,54^ as well as non-neuronal cells^9,33^ with some differences in gene expression reported across male and female donors. In bulk tissue, recent molecular profiling of the hDRG has highlighted differentially accessible chromatin regions (DARs), as well as sex-specific changes in neuropathic pain states at the transcriptome level.^24,44^ To date, the corresponding proteome signature has been missing, as the few prior studies depend on shotgun (data-dependent acquisition) proteomics,^19,49^ which is known to be biased towards high-abundant proteins.^26^

In this study, we generate a quantitative hDRG proteomic dataset across male and female donors using data-independent acquisition (DIA) mass spectrometry. This comprehensive dataset provides a human-centric reference for protein expression in a key structure of the peripheral nervous system and complements previously published assay for transposase-accessible chromatin with sequencing (ATAC-seq) and RNA-seq data. Through integration across omics from the same tissue, we show strong evidence for sexual dimorphism in the tumor necrosis factor alpha (TNFα) signalling pathway. Genome-wide association studies provide support that it is a functionally relevant pathway in the periphery, and evidence from clinical trials highlights a link to clinical sexual dimorphism in response to medication against these targets. The functional relevance of TNFα signalling in the hDRG was confirmed in vitro, and downstream changes in protein phosphorylation suggest a possible mechanism of action. Together, these findings speak to a sexually dimorphic pathway in the peripheral nervous system, which is of particular importance to sensory- and pain-related translation.

## 2. Methods

### 2.1. Dorsal root ganglia tissue procurement

All human tissue procurement procedures were approved by the Institutional Review Board at the University of Texas at Dallas. Through collaboration with the Southwest Transplant Alliance, human lumbar DRGs (hDRGs, L1-L4) from organ donors were obtained within 4 hours of cross-clamp. Tissue for mass spectrometry was frozen in dry ice right immediately and stored in a −80°C freezer. All transport was done on large amounts of dry ice to protect tissue integrity. Age-matched male and female samples of mixed ethnicity were used for this study. Donors for proteomic profiling were negative for pain, neuropathy, and illicit drug use (excluding marijuana), based on recorded patient history.

For fluorescence-activated cell sorting (FACS), hDRGs (lumbar and thoracic) were recovered and stored in 10 mL of Hibernate A (BrainBits HACA500, Thermo Scientific, Massachusetts, USA) supplemented with 1% N-2 (Thermo Scientific, Massachusetts, USA, 17502048), 2% NeuroCult SM1 (Stemcell Technologies, Vancouver, Canada, 05711), 1% penicillin/streptomycin (Thermo Fisher Scientific, Massachusetts, United States, 15070063), 1% Glutamax (Thermo Scientific, Massachusetts, USA, 35050061), 2 mM Sodium Pyruvate (Gibco, Massachusetts, USA, 11360-070), and 0.1% bovine serum albumin (Biopharm, Bluffdale, USA, 71-040) at 4°C until ready for processing (10-16 hours, see below).

### 2.2. Dorsal root ganglia tissue preparation for mass spectrometry

Dorsal root ganglia were processed sequentially, with experimenters blinded to a randomized order (corresponding to the donor column, Table 1). Moreover, 12 to 24 hours before dissection, tissue was transferred to −20°C. For each hDRG, tissue was thawed on ice for ∼15 minutes before dissection (on ice) to remove the surrounding connective tissue. In each ganglia, a region of nerve tissue extending from the core ganglia was identified. This was presumed to contain fewer/no neuronal soma and was transected crudely with a scalpel for separate processing (as the “nerve root” region).

**Table 1:** Donor details.

Donor	Sex	Age	DRG	Pain	HTN	Ethnicity	COD
MS-5	F	32	L2	False	False	Black	Head trauma/GSW
MS-3	F	41	L3	False	True	White	Head trauma/motorcycle accident
MS-7	F	41	L2	False	True	White	CVA/stroke
MS-2	F	55	L3	False	True	White	Anoxia/cardiovascular
MS-1	M	33	L3	False	True	White	Head trauma/MVA
MS-8	M	41	L4	False	True	Black	Head trauma/GSW/suicide
MS-4	M	43	L3	False	False	Hispanic	Head trauma/MVA
MS-6	M	53	L1	False	False	Hispanic	Anoxia/seizure
FACS-1	M	34	2xL3 + T8	False	False	Black	Anoxia
FACS-2	M	37	L1-3, L5	True	False	White	Head trauma/non-MVA
FACS-3	M	31	L3	False	False	Asian	Anoxia
FACS-4	F	29	L1 + L3	False	True	White	Sepsis
FACS-5	F	41	L3-L5	NA	NA	NA	NA

COD, cause of death; CVA, cerebrovascular accident; FACS, fluorescence-activated cell sorting; GSW, gunshot wound; HTN, hypertension; MS, mass spectrometry; MVA, motor vehicle accident.

### 2.3. Protein extraction and SP3-assisted digestion

Owing to the size of the ganglia, each ganglia was cut into up to 4 pieces, and subsequently, each piece was cut into smaller pieces to increase the surface area for lysis. All small pieces of 1 big cut were transferred to a 2-mL LoBind Protein Eppendorf tube (Eppendorf, Hamburg, Germany) prefilled with lysis buffer (2% SDS, 100 mM Tris, 5% glycerol, 10 mM DTT, and 1x protease inhibitor cocktail) and sonicated for 15 minutes (cycles of 30 seconds ON and 30 seconds OFF, 4°C, low frequency) in a Bioruptor Pico (Diagenode, Seraing, Belgium). Samples were subsequently incubated for 15 minutes at 70°C, 1500 rpm in a ThermoMixer C (Eppendorf, Hamburg, Germany) followed by a centrifugation for 5 minutes with 10,000*g* at room temperature (RT) to pellet cell debris. The supernatant was mixed with a 5× sample volume of 100% acetone (prechilled at −20°C) and incubated for 2.15 to 2.45 hours to precipitate the proteins.

Precipitates were pelleted by centrifugation for 30 minutes at 14,000*g*, RT. Pellets were washed with ice-cold 80% EtOH and centrifugated again with the above-mentioned parameters. The EtOH was removed and pellets were air-dried for approximately 20 minutes at RT. Resolubilization in lysis buffer was facilitated by incubation for 10 minutes at 70°C, 1500 rpm. Total protein concentration was determined at 280 nm with a NanoPhotometer N60 (Implen, Munich, Germany). Subsequently, samples were flash-frozen and stored at −20°C until further usage.

Protein clean-up and digestion were then performed as described by Xian et al. (2022). This is based on the single-pot, solid-phase-enhanced sample preparation (SP3) method from Hughes et al. (2019) and is also detailed in the corresponding protocol at protocol.io.^5^ The remaining protein was used for a targeted phosphorylation array (below).

### 2.4. Tandem mass spectrometry

Nanoflow reversed-phase liquid chromatography (Nano-RPLC) was performed on NanoElute2 systems (Bruker Daltonik, Bremen, Germany). This was coupled with timsTOF HT (Bruker Daltonik) through CaptiveSpray ion source. Mobile phase A consisted of 100% water and 0.1% formic acid, and mobile phase B consisted of 100% acetonitrile and 0.1% formic acid.

Five hundred nanograms of peptides were loaded onto a C18 trap column (1 mm × 5 mm, ThermoFisher) and further separated over a 90-minute gradient on an AuroraTM ULTIMATE column (25 cm × 75 µm) packed with 1.6-µm C18 particles (IonOpticks, Fitzroy, Australia). The flow rate was set to 250 nL/minute, except for the last 7 minutes, where the flow rate was accelerated to 400 nL/minute. The mobile phase B was linearly increased from 2% to 20% in the first 60 minutes, followed by another linear increase to 35% within 22 minutes and a steep increase to 85% in 0.5 minutes. Then, a flow rate switch to 400 nL/minute was achieved in 0.5 minutes and was maintained for 7 minutes to the end of the gradient to elute all hydrophobic peptides.

The samples were analyzed in data-independent acquisition (DIA) mode coupled with parallel accumulation serial fragmentation (PASEF). Precursors with m/z between 350 and 1200 were defined in 13 cycles (either 2 or 3 quadrupole switches per cycle) containing 34 ion mobility steps within the ion mobility range of 0.65 to 1.35 (1/k0) with a fixed isolation window of 25 Th in each step. The acquisition time of each DIA-PASEF scan was set to 100 milliseconds, which led to a total cycle time of around 1.48 seconds. The collision energy was ramped linearly from 65 eV at 1/k0 = 1.6 to 20 eV at 1/k0 = 0.6.

### 2.5. Spectral deconvolution with DIA-NN

DIA-NN (version 1.8.1) was used to process raw spectra in library-free mode through the command line on the Vienna Scientific Cluster.^16,17^ A predicted library search was performed against the human proteome (UP000005640) with match-between-runs (MBR) enabled. Separate MBRs were performed for quality control samples, and ganglia + nerve root, as well as for ganglia and nerve root independently when used for differential expression testing. Gene group outputs are also referred to as “proteins” throughout. See the data availability statement for access.

### 2.6. Basic gene lists

Curated lists of pain genes have been previously published.^35,39,63^ Ion channels were matched by Alexander et al. (2023)^2^, and GO-term-related gene lists were extracted through R using biomaRt.^20^ Drug targets were extracted from opentargets.com for relevant conditions.^41^ Receptor types were derived from a previously published interactomics resource^57^ and were compared with published bulk RNA-seq from the DRG.^44^

### 2.7. Gene set enrichment analysis

Gene set enrichment analysis (GSEA) for neuronal subtype enrichment was performed against gene lists derived from Zheng et al.,^64^ for mouse subpopulations as described previously.^7^ In brief, RNA-seq count data from GSE131230—which had been processed using STAR alignment and HTSeq on the same genome build (see Zheng et al., for full methods)—was corrected for library size and transformed using rlog in R with DESeq2.^37,65^ This was then filtered to match their published report. Genes with an average rlog above the 95% quantile cut-off per subpopulation were curated into a “gene set” for enrichment. Mouse gene names were converted using the biomaRt package through the ensembl mart with “getLDS().”^20^

For human subpopulations as described in Taveres-Ferreira et al.,^54^ marker genes were accessed directly from the supplemental tables and reformatted in R for processing. In brief, these genes were selected using the “findMarkers()” function in Seurat after visium-based spatial sequencing.

These custom gene sets were then compiled for a GSEA analysis using the clusterProfiler package.^60^ Minimum gene set size was set to 25 (no maximum size threshold) and run for 10,000 permutations.

The average expression across ganglia samples was calculated per gene group after filtering for 80% completeness. Where the first term per gene group overlapped (eg, TRPV1 vs TRPV1, TRPV2, TRPA1), the first term in the group was used, and the row with the higher abundance was taken. Gene set enrichment analysis was then performed on the ranked mean expression.

To examine pathway differences between male and female samples, GSEA was performed on ranked log2 fold changes (LFC) output with a false discovery rate (FDR) < 0.05; minimum gene set size was set to 25. Gene sets were extracted using the “msigdbr” package in R.^18^ Hallmark pathways were extracted as “category = ‘H.’” Biological pathways (BPs) and molecular function (MF) from gene ontology (GO) lists were extracted as category = “C5,” subcategory = “BP” and category = “C5,” subcategory = “MF,” respectively.

For the proteomics data, LFC were calculated using limma (see hypothesis testing). For GSEA on RNA-seq, DESeq2 was used to calculate LFC, as described in Farah et al., (2024)^23^, using previously published bulk RNA-seq data (n = 31 M, 19 F donors).^44^ For ATAC-seq, LFC were derived from pseudobulk analysis of all clusters in a spatial-seq dataset in autosomes, described by Franco-Enzástiga et al., (2024). In brief, ATAC-seq gene scores were calculated using ArchR. The gene score predicts the expression of genes based on the chromatin accessibility of regulatory elements in the vicinity of a gene and its corresponding gene body. In ArchR, the function “GeneScoresMatrix()” was used to store gene scores. To sum together gene scores of all the cells from the same sample for the pseudobulk analysis, the “getMarkerFeatures()” function in ArchR was used, and the “groupBy = ‘Sample’” was specified. Data were compared across sex (n = 5 M, 3 F donors).

### 2.8. Overrepresentation analysis

Overrepresentation analysis was performed using the “enrichGO” function from clusterProfiler.^60^ Differentially expressed proteins (DEPs, abs [LFC] > 1, FDR < 0.05) were compared with a background of gene groups used for the initial limma comparison using default settings.

### 2.9. Supervised PCA

Supervised principal component analysis (sPCA)^3^ was performed as previously described.^7^ Differentially expressed genes (DEGs) from spatial RNA-seq and differentially expression regions (DARs) from bulk hDRG ATAC-seq datasets exploring sexual dimorphism were extracted as lists.^24,54^ DARs were filtered to remove X and Y chromosome regions. Differentially expressed genes from Taveres-Ferreira et al. (2022) were extracted from supplemental Table 2, http://links.lww.com/PAIN/C301, “B-Overall_neurons_DE_genes” looking at differential expression between male and female barcodes within neurons.

Proteomic data from the ganglia (merged by replicate) was subset by either DEGs or DARs and subject to a PCA (using “prcomp” in R). Eigengenes and eigenvectors were then extracted from the first principal component (“PC1”).

### 2.10. Hypothesis testing

Hypothesis testing was performed within ganglia samples using a moderated *t* test with limma,^45^ modelling for sex, n = 4 M and 4 F donors. In this study, log2 transformed data were first filtered for the primary occurrence of each gene group (ie, protein) and filtering out proteins present in less than 80% of samples. For example, where gene group A = “TRPV1” and B = “TRPV1, TRPV2, TRPA1,” gene group A would be used. *P* values were corrected with Benjamini-Hochberg, and a significance threshold was set to FDR < 0.05, LFC >1.

### 2.11. Multi-study factor analysis

Strong correlations between ATAC-seq regions, RNA-seq transcript abundance, and protein abundance were not evident. Even so, matching sexually dimorphic pathways are described across datasets. In this study, we employed a multi-study factor analysis^56^ to examine latent variables in the context of sex to look for the underlying structure contributing to this overlap.

Following the principles for factor analyses on small sample sizes,^58^ we limited our search to a small number of factors (here, 3) and used sexually dimorphic DARs. This allows us to look exclusively at chromatin regions implicated in female/male differences: This biases the analysis towards factors involved in sexual dimorphism and provides a clear link from ATAC-RNA-protein. The within-dataset factor numbers were determined by scree plots. A minimum Kaiser-Meyer-Olkin (KMO) threshold was set to 0.5 for each dataset, and only sex-related factors were considered from the output (ie, a confirmatory model, as opposed to an exploratory factor analysis). Owing to the arbitrary nature of factor loading signs, within-dataset factor loadings were constrained such that they were positively correlated to men if sex appeared relevant.

Bulk RNA-seq from human DRG^44^ were extracted as quantile-normalized transcripts per million (qnTPM) for neuronal-enriched donor samples (n = 50). Both “pain” and “nonpain” donors were considered here to examine sexual dimorphism across states. Proteomic samples from the “ganglia” and “nerve root” regions of the DRG were used and merged across technical replicates by mean. In this study, we acknowledge the limitation that this is a low sample number and that samples are paired (16 samples from 8 donors). We limited this effect by analyzing only factors shared across 2 datasets, specifically in the context of sex, and not within the proteomic dataset in isolation.

Genes were matched to bulk DARs from Franco-Enzástiga et al. (2024) and filtered to remove genes with missing values across both datasets, resulting in complete data matrices. Data were then transposed for multi-study factor analysis (RNA = [50 × 402], proteomics = [16 × 402]). Starting values were extracted using “start_msfa,” constraint = “block_lower2,” capped at 10,000 iterations. “ecm_msfa” was then run with default parameters.

Factor scores were estimated using the weighted least squares (ie, the Bartlett Method; Equation 1), where X∼ is the centred/scaled data matrix for each dataset, *i*. Lambda are the factor loadings shared across datasets and Psi is a diagonal matrix equal to the specific variances.

(1) $${\hat{f}}_{i}={\left(\Lambda \Lambda^{T}\psi^{-1}\right)}^{-1}\Lambda^{T}\psi^{-1}\left(\tilde{X_{i}}\right)$$

The interaction of “Sex” and “Factor” was tested by an ANOVA. Post hoc testing was then performed per factor (eg, Male.Factor1 − Female.Factor1) using “glht (anova, linfct = mcp (Interaction))” in R. Factors with a q < 0.1 were considered significant. Shared gene loadings were extracted from the multi-study factor analysis output as phi for visualization.

Gene set enrichment analysis analyses were performed as above, with the following modification: minimum gene set size set to 5 (due to the total number of genes available, 402). In line with the above, the significance threshold was set to FDR < 0.05. Protein-protein interaction networks were plotted through https://livedataoxford.shinyapps.io/drg-directory/,^63^ which uses an API with STRING DB and overlays sensory neuron and pain-relevant datasets through R Shiny.

### 2.12. Fluorescence-activated cell sorting preparation

The DRGs were trimmed of excess connective tissue, fat, and nerve roots to collect the bulb-containing neuronal cell bodies. The bulb of each DRG was cut into 3 mm sections and placed in 5 mL of prewarmed digestion enzyme containing 2 mg/mL of Stemxyme I (Worthington Biochemical, Lakewood, USA, LS004106), 10 ng/mL of recombinant human β-NGF (R&D Systems, Minnesota, USA, 256-GF), and 0.1 mg/mL of DNAse I (Worthington Biochemical, Lakewood, USA, LS002139) in HBSS without calcium and magnesium (Thermo Scientific, Massachusetts, USA, 14170-112).

The tubes were placed in a 37°C shaking water bath and triturated every hour until the DRG sections were dissolved (3-4 hours). Samples were filtered through a 70-µm mesh strainer and centrifuged at 350*g* for 5 minutes at room temperature (all subsequent centrifuge steps follow the same parameters). The supernatant was removed, and the pellet was resuspended in a red blood cell lysis buffer (Biolegend, San Diego, USA, 420301) and incubated at room temperature for 5 minutes. Samples were then centrifuged, the supernatant was removed, and the pellet was resuspended in 0.5% bovine serum albumin in 1X phosphate-buffered saline (PBS). To remove myelin from the dissociation, cells were incubated with myelin removal beads (Miltenyi Biotec, Gaithersburg, USA, 130-096-433) for 15 minutes at room temperature. Cells were washed with 1 mL of 0.5% bovine serum albumin in PBS, spun down, resuspended in 0.5% bovine serum albumin in PBS, and passed through an LS column (Miltenyi, 130-042-401) on a MidiMACS separator (130-042-301) according to manufacturer's protocol. Samples were then spun down and resuspended in PBS and proceeded to cell staining.

Cells were stained with a fixable live/dead stain (Biolegend, San Diego, USA, 423107) for 10 minutes at room temperature and protected from light. Cells were washed with 1 mL of flow cytometry staining buffer (Invitrogen, 00-4222-26), spun down, and resuspended in flow buffer. Cells were incubated with an Fc receptor blocker (TruStain FcX, Biolegend, 422302) for 10 minutes at room temperature, protected from light. Cells were then incubated with CD45, CD11b, and CD3 antibodies for 30 minutes on ice, protected from light (see Supplemental Table 10, http://links.lww.com/PAIN/C301 for antibodies used in flow cytometry experiments). Cells were washed with 1 mL of flow cytometry staining buffer, spun down, resuspended in flow buffer, and kept on ice until processing. Fluorescently activated cell sorting was used to isolate Live, CD45^+^, CD11b^+^, and CD3^−^cells on a BD FACS Aria Fusion (Gating Strategy in Supplemental Fig. 9A, http://links.lww.com/PAIN/C300). Cells were collected, spun down, and resuspended in prewarmed RPMI media (Gibco, 11875-093) containing 10% HyClone Fetal Bovine Serum (Thermo Fisher Scientific, SH3008803IR) and 1% penicillin/streptomycin. Cells were plated at 20k cells per well in a 96-well plate and allowed to acclimate overnight in an incubator (37°C, 5% CO_2_). All antibodies are listed in Supplemental Table 10, http://links.lww.com/PAIN/C301.

### 2.13. Lipopolysaccharide stimulation of human dorsal root ganglia immune cells

Cells were stimulated with lipopolysaccharide (LPS) (10 ng/mL, Sigma-Aldrich, L6529) or its vehicle (RPMI media) for 16 hours, as this dose has previously been shown to drive increased secretion of TNFα from human myeloid cells in vivo.^1,11^ In 1 condition, cells were co-stimulated with Brefeldin A (1:1000, Biolegend, 420601). In cultures without Brefeldin A, the cell culture supernatant was collected and frozen at −80°C till processing. TNFα levels in the media supernatant were measured with a Legendplex kit targeting TNFα (Biolegend, San Diego, USA, 741187), per the manufacturer's recommended protocol. The average of 2 technical replicates was used to calculate TNFα concentrations. In cells co-cultured with Brefeldin A, cells were collected and fixed with a Cyto-Fast fixation and permeabilization kit (Biolegend, 426803) for 20 minutes at room temperature. Cells were washed twice with the Cyto-Fast perm wash solution and then incubated with a TNFα antibody for 30 minutes on ice. Cells were washed with 1 mL of flow cytometry staining buffer, spun down, resuspended in flow buffer, and kept on ice until data acquisition on a BD LSRFortessa. The percentage of cells expressing TNFα was calculated for each condition using FlowJo (Version v10.10) (Gating Strategy in Supplemental Fig. 9B, http://links.lww.com/PAIN/C300).

### 2.14. TNFα stimulation

Human dorsal root ganglia immune cells were stimulated with TNFα (10 ng/mL, R&D Systems, 210-TA) or its vehicle (1X PBS) for 30 minutes. This dose and timepoint have been shown to increase p38 levels in human cultures of myeloid cells and fibroblasts.^29,53^ Cells were then collected and fixed with 4% paraformaldehyde (Electron Microscopy Sciences, Hatfield, USA, 15710) in PBS for 15 minutes at room temperature. Cells were then washed 2× with flow cytometry staining buffer and permeabilized with prechilled, 100% methanol (Fisher Scientific, A456-212), for 30 minutes on ice. Cells were then washed 2× with flow cytometry staining buffer and incubated with p38 and p65 antibodies in flow buffer for 30 minutes on ice. Cells were washed with 1 mL of flow cytometry staining buffer, spun down, resuspended in flow buffer, and kept on ice until processing. The percentage of cells expressing p38 and p65 along with the median fluorescence intensity of each signal was calculated for each condition (Gating Strategy in Supplemental Fig. 9B, http://links.lww.com/PAIN/C300). The median fluorescence intensity values were normalized to an unstained control.

### 2.15. Targeted phosphorylation array

Phosphorylation levels were measured in remaining hDRG protein samples using the Proteome Profiler Human Phospho-Kinase Array Kit (R&D Systems, CAT# ARY003C). The commercial protocol was followed, with the following changes: Each membrane was washed independently using 7 mL Wash Buffer in 60 × 15 mm culture dishes, and membranes were rinsed 1× with wash buffer after antibody incubation before 3 × 10 minutes washes. A ChemiDoc MP Imaging Platform (Bio-Rad, Feldkirchen, Germany) was used to detect signals. Four samples per condition were processed for membrane “A,” while 3F + 4M were processed for membrane “B.”

In brief, the protocol is as follows: Membranes were blocked with “Array Buffer 1” for 1 hour at RT before an overnight incubation at 4°C with 250 µg protein per membrane (ie, 500 µg per membrane pair, A&B), each diluted to 1 mL in “Array Buffer 1.” Membranes were then washed (3 × 10 minutes), incubated with membrane-specific antibodies for 2 hours at RT, rinsed 1× before washing (3 × 10 minutes), and incubated with Streptavidin-HRP for 30 minutes at RT. After washing (3 × 10 minutes), membranes were incubated for 90 seconds with the “Chemi Reagent Mix,” blotted with Kimwipes, and imaged on a ChemiDoc MP Imaging Platform for 240 seconds. All membranes per round (2 M/2 F per round) were developed and imaged together.

Image quantification was performed in ImageJ. Regions of Interest (ROIs) of equal size were processed using the ROI Manager, and quantification was considered as x̄_(pixel intensity, PBS)_ − x̄_(pixel intensity, ROI)_ for each phosphorylation site, using the PBS negative control per membrane as the background.

### 2.16. Graphics

All plots were generated in R with the following libraries unless otherwise described: ggplot2, ComplexHeatmap, cowplot, gridExtra, ggbiplot, and ggrepel. Figures were compiled in Inkscape.

## 3. Results

**Figure 1.**
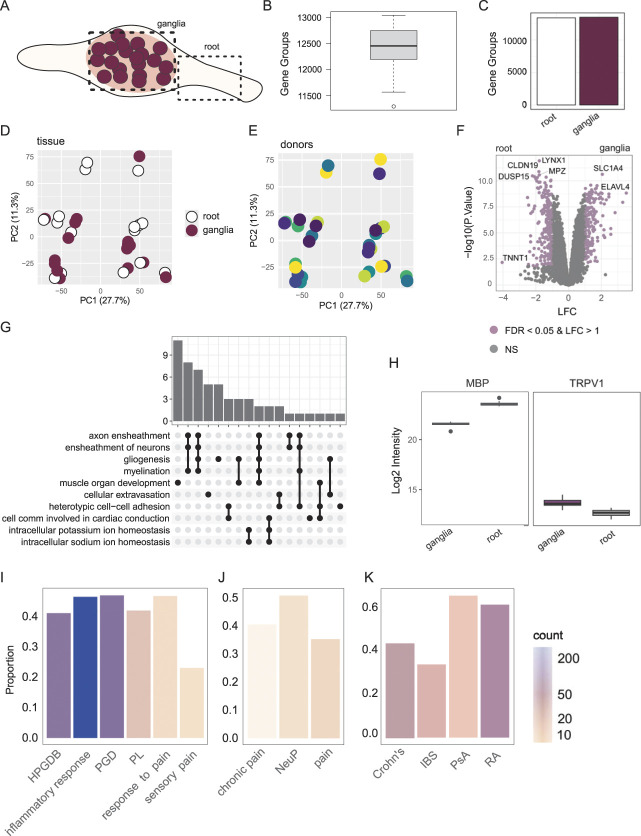
Quantitative proteomics of the hDRG and nerve root. (A) Schematic of the ganglia and nerve root (NR, root). (B) Gene groups (protein counts) per sample. (C) Gene group count per tissue. (D, E) PCA for tissue (D) and donor (E). (F) Volcano plot of differentially expressed proteins (DEPs, ganglia vs nerve root; positive LFC for ganglia), coloured by abs (LFC) and FDR < 0.05. NS, not significant. (G) Upset plot showing GO term over enrichment analysis for differentially expressed proteins upregulated in the nerve root. (H) Example DEPs for myelin basic protein (MBP) and TRPV1 (adjusted *P* < 0.01 each). (I–K) Overlap of detected gene groups with relevant gene sets: related to pain (I), pain drugs (J), and peripheral autoimmune conditions (K). HPGDB, human pain genetics database.^39^ PGD, pain gene database.^35^ PL, DoloRisk priority group pain list.^55^ NeuP, neuropathic pain; IBS, irritable bowel syndrome; PsA, psoriatic arthritis; RA, rheumatoid arthritis. Related to SFig. 1, http://links.lww.com/PAIN/C300. n = 8 donors (4 M + 4F) for ganglia + nerve, with technical replicates (×2 per sample) merged for downstream analyses. hDRG, human dorsal root ganglia.

In this study, we present the most comprehensive proteomic dataset of the human dorsal root ganglia (hDRG) and the nerve root (Fig. 1A), with previous datasets using shotgun proteomics.^19,49^ Using data-independent acquisition coupled with parallel accumulation serial fragmentation (DIA-PASEF) we identify ∼12,500 gene groups (proteins) per sample, with similar levels between the ganglia and root (Figs. 1B and C, see Data Availability). Donor details are available in Table 1. We confirmed data quality using control samples throughout the MS runs for both LC-MS and SP3 replicates (SFig. 1A, http://links.lww.com/PAIN/C300), and do not see overt variance by donor or age (SFig. 1B, http://links.lww.com/PAIN/C300). As expected, samples show the highest correlation within sample replicates (SFig. 1C, http://links.lww.com/PAIN/C300). Even so, samples do not cluster obviously by tissue type or donor (Figs. 1D and E) or by sex/replicate (SFig. 1D, http://links.lww.com/PAIN/C300), suggesting a generalized proteome signature across all samples. These technical replicates were merged (by mean), with 32 samples (16 × 2 replicates) averaged to 8 ganglia + 8 nerve samples for downstream analyses (from 4 M + 4 F donors).

When comparing the nerve root to the ganglia, we see large differences in protein (aka “Gene Group”; see methods) levels. These differentially expressed proteins (DEPs) show a bias in myelin-associated terms upregulated in the root and soma-related terms in the ganglia (Figs. 1F–H). A full list of DEPs is provided in Supplemental Table 1, http://links.lww.com/PAIN/C301, with GO enrichment in Supplemental Tables 2-3, http://links.lww.com/PAIN/C301. This is expected, given our knowledge of these tissues, and gives confidence to this dataset to probe other questions. With our current dataset quality and coverage, we see this data as a reference for the hDRG proteome, with extensive coverage of relevant curated gene lists for pain targets (Fig. 1I), pain-related drug targets (Fig. 1J), and peripheral autoimmune targets (Fig. 1K).

**Figure 2.**
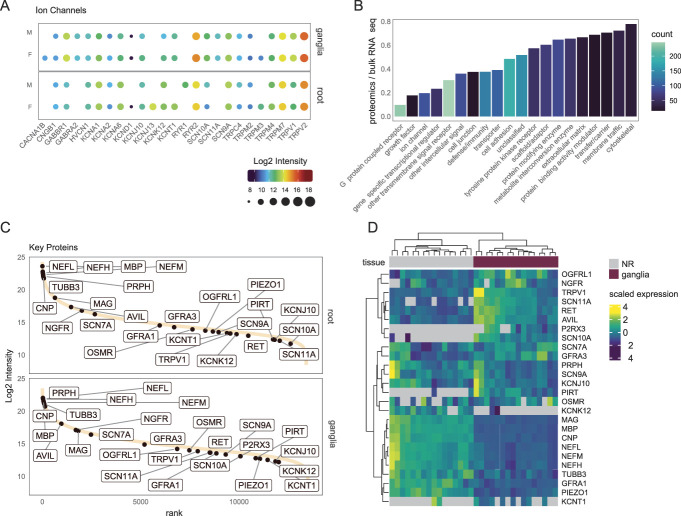
Ion channel and G-protein coupled receptor (GPCR) expression across tissues. (A) Average expression by ion channel across tissues. (B) Proportion of receptor detection in the proteomics data vs bulk RNA-seq of the hDRG.^44^ (C) Dynamic range plot showing ranked intensity across tissues. Key proteins (gene groups) for sensory neurons and myelin are highlighted. (D) Heatmap across all samples (including each technical replicate) highlighting the dataset completeness for key proteins in (C). n = 8 donors (4 M + 4F), with samples for ganglia + nerve; each sample has a technical replicate. hDRG, human dorsal root ganglia.

Given the importance of membrane proteins in neurological and neuroimmune conditions in the PNS, as well as the previous difficulties in detecting membrane proteins using mass spectrometry, we specifically investigated ion channel and receptor abundance in our dataset (Fig. 2).^49^ In this study, we detect a number of TRP-, SCN-, and KCN-ion channels (Fig. 2A), among others, as well as a small number of GPCRs (Fig. 2B). These candidates are expressed throughout our dynamic range of protein abundance (Fig. 2C), with a number of key proteins detected almost exclusively in the ganglia (eg, P2RX3, SCN10A, Fig. 2D). In addition, when we compare these data to a curated list of proteins for sensory neurons and myelin, we see a clear separation of tissue types, again highlighting the strength of this dataset (Fig. 2D).

### 3.1. The ganglia and nerve root

**Figure 3.**
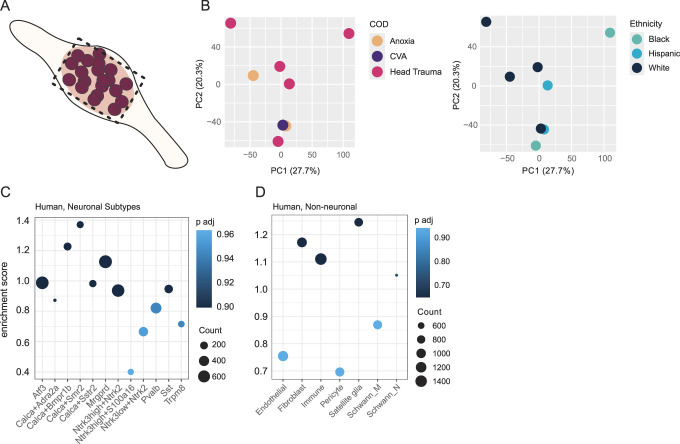
DIA-PASEF proteomics of the hDRG has markers across cell types. (A) Schematic. (B) PCA, coloured by cause of death (COD, left) and ethnicity (right). (C) Gene set enrichment analysis (GSEA) against human neuronal subtype markers. (D) GSEA against non-neuronal cell type markers. Related to SFigs. 2–3, http://links.lww.com/PAIN/C300. n = 4M + 4F donor DRGs. DIA-PASEF, data-independent acquisition-parallel accumulation serial fragmentation; hDRG, human dorsal root ganglia.

Because of the differences between the ganglia and nerve root (Figs. 1F–H), we independently investigated the proteome of each tissue type. In the ganglia (Fig. 3A), we again do not see obvious clustering by PCA over the cause of death and ethnicity (Figs. 3B and C). The samples cluster by replicate (SFig. 2A, http://links.lww.com/PAIN/C300) and show consistent protein counts (SFig. 2B, http://links.lww.com/PAIN/C300). In line with previous reports for quantitative proteomics, the data also does not correlate strongly to bulk RNA-seq of the same tissue,^44^ with an R^2^ = 0.15 (SFig. 2C, http://links.lww.com/PAIN/C300). When looking more precisely at receptor types, we see a highly variable correlation across receptor types, ranging from -0.2 to 0.8 (SFig. 2D, http://links.lww.com/PAIN/C300), likely reflecting a combination of biological and technical differences in receptor type proteomics, due in part to regulatory dynamics.

Next, we examined the enrichment profiles of neuronal and non-neuronal cells using custom genesets derived from snRNA-seq^9^ (Fig. 3D, see methods). In this study, we do not see a specific significant enrichment for any cell type but instead can detect marker genes across populations. This trend is mirrored when using gene sets derived from mouse datasets^64^ (SFig. 3A-B, http://links.lww.com/PAIN/C300), and for neurons using a secondary, visium-based, hDRG dataset^54^ (SFig. 3C, http://links.lww.com/PAIN/C300).

**Figure 4.**
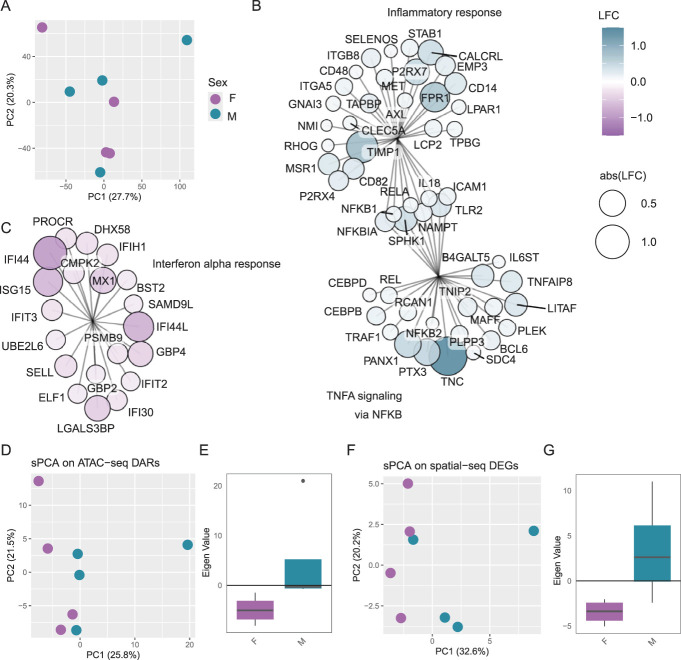
Sexual dimorphism in the hDRG. (A) PCA of ganglia samples (merged by replicate). (B–C) Representative gene set enrichment analysis (GSEA) pathway enrichments (male = positive, female = negative LFC). (B) TNFα signalling through NFκB with overlap to inflammatory response. (C) IFNα response. Supervised PCA (sPCA) and corresponding eigenvalues (PC1) of ganglia samples on DARs (ATAC-seq)^24^ (D, E), and DEGs (RNA-seq).^54^ Related to SFigs. 4–7, http://links.lww.com/PAIN/C300. n = 4M + 4F donor DRGs. DAR, differentially accessible chromatin region; DEG, differentially expressed genes; hDRG, human dorsal root ganglia; IFNα, interferon α; NFκB, nuclear factor kappa B.

We then set out to investigate male/female differences in the hDRG (Fig. 4, SFig. 4, http://links.lww.com/PAIN/C300). Ganglia samples do not cluster by sex (Fig. 4A) and no differentially expressed proteins were detected between men and women (SFig. 4A, http://links.lww.com/PAIN/C300, Supplemental Tables 4-5, http://links.lww.com/PAIN/C301). Even so, GSEA highlights pathway differences (Figs. 4B and C, SFig. 4B–D, http://links.lww.com/PAIN/C300). Notably, TNFα signalling is enriched in male donors (Fig. 4B), while interferon (IFN) α response (Fig. 4C) and oxidative phosphorylation (SFig. 4D, http://links.lww.com/PAIN/C300) are enriched in female samples.

To see if there was a shared signature of sexual dimorphism across omics types (ie, ATAC-seq and RNA-seq), we next clustered the ganglia samples by sPCA against previously published differentially accessible regions (DARs)^24^ and DEGs,^54^ comparing hDRG of male and female donors (Figs. 4D–G).

We see a separation of male and female proteomic samples by sPCA against sexually dimorphic DARs (ATAC-seq, Figs. 4D and E) and DEGs (RNA-seq, Figs. 4F and G), although this trend is not statistically significant (Welch, *P* ∼ 0.1) in either case. PCA and sPCA are dimensionality reduction techniques that calculate a covariance matrix to represent high-dimensional data (eg, many proteins) in fewer dimensions (eg, the first and second principal components), with the components optimized to maximize variance. From this, the contribution of each protein to each principal component can still be extracted. In this study, eigengenes were extracted from the first principal component to see what was driving this separation by sex, with a number of proteins in the TNFα signalling pathway showing high loading values, suggesting they contribute to driving the difference across sex (SFig. 5, http://links.lww.com/PAIN/C300).

To see if the pathway changes from our proteomic data are shared across omics, we next performed GSEA against pseudobulk spatial ATAC-seq (SFig. 6A, http://links.lww.com/PAIN/C300), as well as bulk RNA-seq from thoracic vertebrectomy participants (SFig. 6B, http://links.lww.com/PAIN/C300).^24,44^ TNFαsignalling through nuclear factor kappa B (NFκB) was enriched in men across datasets, suggesting a cross-omic pattern of sexual dimorphism.

The nerve root (SFig. 7A, http://links.lww.com/PAIN/C300) exhibits slightly fewer protein identifications (SFig. 7B, http://links.lww.com/PAIN/C300) than the ganglia, but again do not cluster by sex (SFig. 7C, http://links.lww.com/PAIN/C300) or show differentially expressed proteins by sex (SFig. 7D, http://links.lww.com/PAIN/C300, Supplemental Tables 6-7, http://links.lww.com/PAIN/C301). Mirroring the GSEA from Figure 4, we see a different pattern of sexual dimorphism in the nerve root compared with the ganglia. Similar to the ganglia, we see an enrichment for oxidative phosphorylation in female donors but do not replicate the differences in IFNα response or TNFα signalling through NFκB (SFig. 7E, http://links.lww.com/PAIN/C300). By sPCA, we also do not see a clear separation by sex using DARs (SFig. 7F, http://links.lww.com/PAIN/C300) or DEGs (SFig. 7G, http://links.lww.com/PAIN/C300) from the corresponding hDRG ATAC-seq and RNA-seq datasets.^24,54^

### 3.2. Sexual dimorphism across omics datasets

**Figure 5.**
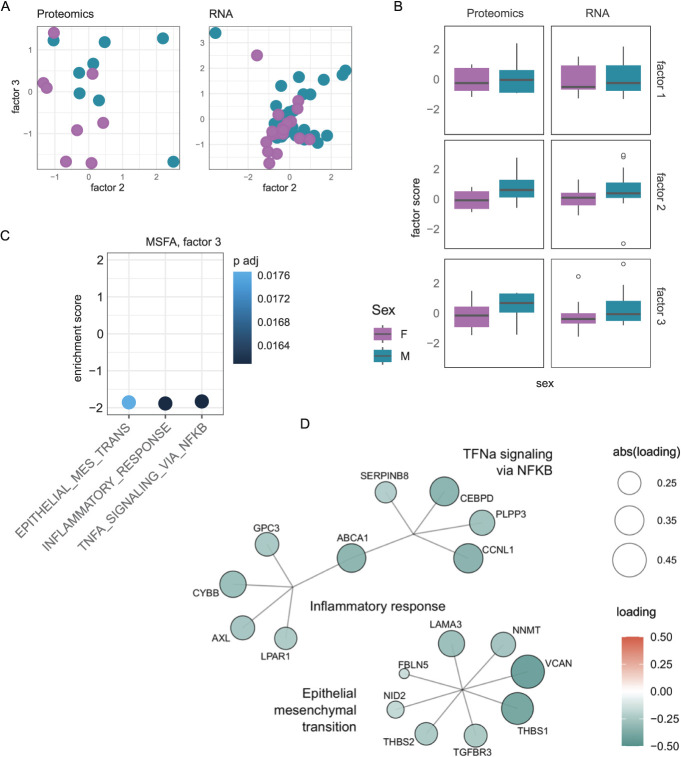
Multi-study factor analysis between bulk RNA-seq and bulk proteomic data, based on differentially accessible regions from bulk ATAC-seq. (A) Scatter plot by factor, for proteomics (left) and transcriptomics (right). (B) Boxplot of factor scores, stratified by sex, bars = ±1.5 × interquartile range. (C) Gene set enrichment analysis (GSEA) on factor 3 (ranked loadings). (D) Pathways + gene loadings from GSEA output of factor 3. Related to SFig. 8, http://links.lww.com/PAIN/C300.

To probe shared patterns of sexual dimorphism across omics types, we used a multi-study factor analysis on available hDRG datasets (Fig. 5, SFig. 8, http://links.lww.com/PAIN/C300).^24,44^ Multi-study factor analysis allows us to examine class separation (here, men and women) through shared factors across omics types, where each relevant factor has interpretable underlying constructs.

We used the most comprehensive bulk RNA-seq dataset available to date, looking at neuropathic pain in a participant cohort of thoracic vertebrectomy patients undergoing surgery. Of the 70 DRG sequenced, 50 showed neuronal enrichment, as described in the original publication.^44^ These 50 DRG, removed during thoracic vertebrectomy surgical procedure, were stratified by sex for comparison with our hDRG proteomic dataset (both ganglia and nerve root). There were too few samples from the hDRG ATAC-seq datasets for a factor analysis; thus, we instead restricted our factor analysis to genes/proteins, which correspond to DARs between male and female donors (see methodology).^24^

Likely because of the small sample sizes, only 3 shared factors were extracted, with factors 2 and 3 both showing a separation by sex (Figs. 5A and B). Gene set enrichment analysis against the ranked loading revealed no hallmark pathway enrichment for factor 2, but 3 enriched pathways for factor 3: TNFα signalling through NFκB, inflammatory response, and epithelial-mesenchymal transition (Figs. 5C and D, SFig. 8A, http://links.lww.com/PAIN/C300). While there are overlapping terms driving TNFα signalling through NFκB and inflammatory response, the enrichment of epithelial-mesenchymal transition is independent of this (Fig. 5D). However, it was also detected in our proteomics data (SFig. 4B, http://links.lww.com/PAIN/C300) and in bulk RNA-seq (SFig. 6B, http://links.lww.com/PAIN/C300).

### 3.3. TNFα release from human dorsal root ganglia immune cells

TNFα signalling consistently appears as a sexually dimorphic pathway in these omics datasets, spanning multiple sets of donors and a large cohort of surgical participants.

If the sexual dimorphism we see is functionally relevant, we would expect a few things: First, drugs targeting the pathway should show response differences in men and women (they do, Table 2). Second, SNPs against core genes in the pathway would be related to pain and sensory processing (they are, Table 3). In this study, sexual dimorphism at the genetic level is also discussed for the TNFα promotor in the context of migraine Fawzi et al., (2015).

**Table 2:** Example clinical outcomes on TNFα inhibitors and sexual dimorphism.

PMID	Citation	Title	Condition	Relevant outcome
Primary examples				
PMID: 19815494	[10]	Effectiveness of adalimumab in treating patients with active psoriatic arthritis and predictors of good clinical responses for arthritis, skin, and nail lesions	Psoriatic arthritis	Male sex increased the chance of achieving a good clinical response
PMID: 28762060	[12]	A 2-year observational study on treatment targets in psoriatic arthritis patients treated with TNF inhibitors	Psoriatic arthritis	Women less likely to achieve remission
PMID: 16705046	[30]	Predictors of response to anti-TNF-α therapy among patients with rheumatoid arthritis: results from the British Society for Rheumatology Biologics Register	RA	Women less likely to achieve remission
PMID: 23322995	[62]	Sex-dimorphic adverse drug reactions to immune suppressive agents in inflammatory bowel disease	IBD	Higher ADR in women taking TNFα-inhibitor
PMID: 31310690	[48]	Earlier discontinuation of TNF-α inhibitor therapy in female patients with inflammatory bowel disease is related to a greater risk of side effects	IBD	Higher ADR in women taking TNFα-inhibitor
PMID: 12492735	[14]	Infusion reactions to infliximab in children and adolescents: frequency, outcome, and a predictive model	Crohn	Female sex as a predictor for side effects in TNFα-inhibitors for Crohn
Example reviews				
PMID: 37820857	[13]	Sex-oriented perspectives in immunopharmacology	Mixed	TNFis show “higher efficacy and adherence in males, More frequent serious infections in males, more frequent toxic liver disease and lupus-like syndrome in women”
PMID: 29754330	[46]	Gender differences in axial spondyloarthritis: women are not so lucky	Axial spondylo-arthritis	Increased TNF levels in only male patients with AS, treatment efficacy of TNFi is significantly lower in women compared with men with axSpA, and they have a significantly lower drug adherence
PMID: 26490106	[52]	Rate of discontinuation and drug survival of biologic therapies in rheumatoid arthritis: a systematic review and meta-analysis of drug registries and healthcare databases	RA	Female sex as a predictor of discontinuation

IBD, inflammatory bowel disease; RA, rheumatoid arthritis; TMD, temporal mandibular.

**Table 3:** Summary of pain-related genome-wide association studies hits for the TNFα signalling pathway from the human pain genetics database.^39^

PMID	Loci	Variants	Direction	Phenotype	Comments from HPGDB, truncated with […]
PMID:18990769		rs1800629	Up	Analgesia	AKA TNF-308 G/A
PMID:18990769		rs1800629	Up	Analgesia	AKA TNF-308 G/A, patients with this genotype did not show improvement in pain scores between 2 assessment periods
PMID:20035431		rs1800629	Up	Migraine	A borderline association was observed in TNFΑ 308 GA genotype in patients with migraine vs controls […]
PMID:25434717		rs1799724	Up	Migraine	TNF-α-857 CT genotype and T allele were associated with an increased risk of migraine. TNF-α-857 CT genotype was associated with an increased risk of migraine without aura (MO) or with aura (MA) in women or men. *While -857T allele was significantly associated with MO or MA in men and with MA only in women*
PMID:25434717		rs1800629	Up	Migraine	TNF-α-308 GA, AA genotypes, and A allele were associated with an increased risk of migraine. TNF-α-308 GA, AA genotypes, and A allele or AA genotype were associated with increased risk of migraine with aura (MA) and migraine without aura (MO), respectively; *this was more significant in female patients with MA than in male patients*
PMID:25304131	TNF	rs1800610	Up	Cancer pain	Five-fold increase in the odds of having high pain and high fatigue
PMID:25315199	TNF	rs3093664	Down	Migraine	Associated with increased risk of all migraine types and the subgroup of MRM (menstrually related migraine)
PMID:26798969	TNFRSF11B	rs2073618	Up	Cancer pain	Increased risk of pain with aromatase inhibitor therapy for musculoskeletal toxicity in patients with breast cancer
PMID:30795980	TNFRSF11B	rs2073618	Up	TMD disorders	In individuals carrying the CC genotype, there was a 10.80 times greater chance of presenting with TMJ ankylosis […]
PMID:30075559	TNFRSF1A	rs767455	Up	Neuraxial pain	A higher frequency of G allele was observed in the ankylosing spondylitis group compared with the control group […]
PMID:23852407	TNFRSF1B	rs1061622	Up	Cancer pain	Predictive for the symptom cluster of pain, depressed mood, and fatigue in patients with lung cancer
PMID:30075559	TNFRSF1B	rs1061622	Up	Analgesia	Associated with long-term efficacy of etanercept
PMID:19773451	NFΚBIA	rs8904	Down	Cancer pain	Reduced risk of pain in patients with lung cancer
PMID:34450027	TNC	rs1330349	Up	Arthritis	Significantly associated with hip osteoarthritis
PMID:34450027	TNC	rs1330349	Up	Postoperative pain	Significantly associated with total hip replacement pain
PMID:27649267	TLR2	rs3804100	Up	Analgesia	This SNP was associated with reduced morphine use overall […]
PMID:25145994	ICAM1	rs5498	Up	Migraine	[…] polymorphism between migraine cases and controls, and between migraine without aura subtype of migraine cases and control

TMD, temporal mandibular; TNFα, tumor necrosis factor alpha.

**Figure 6.**
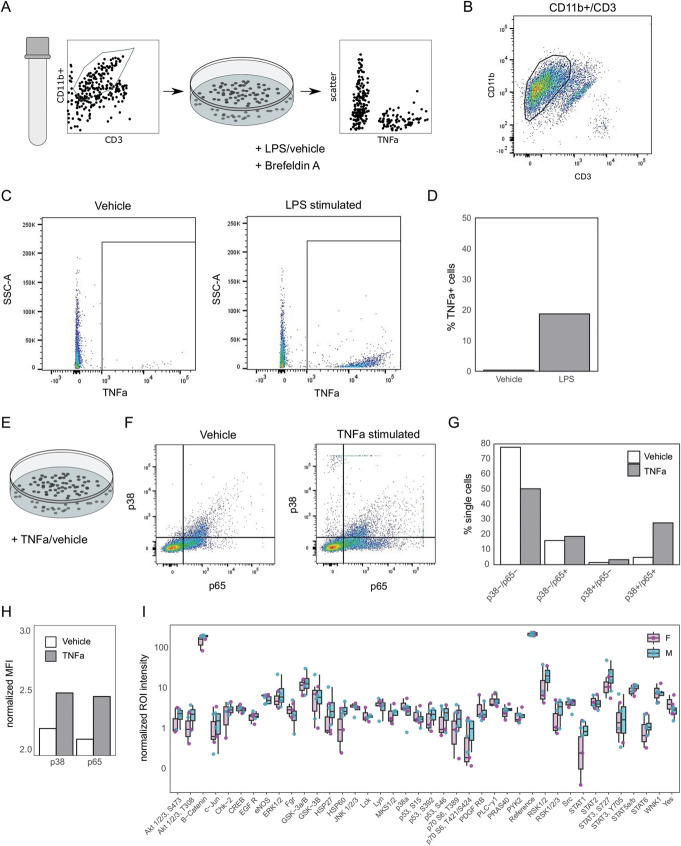
TNFα signalling in the hDRG. (A) FACS experiment overview, for isolation and culturing of myeloid cells followed by stimulation in vitro (n = 1 donor, man). (B) Myeloid cell gating strategy, full strategy shown in SFigure 9, http://links.lww.com/PAIN/C300. (C) Representative flow cytometry for vehicle (left) and LPS (right) stimulation of myeloid cells. (D) Percentage of TNFα+ single cells after LPS stimulation. (E) Schematic for TNFα stimulation of myeloid cells. (F) Transcription factor phosphorylation (p38 and p65) from vehicle (left) and TNFα (right) treated cells. (G) Quantification of (F), % of single cells per condition/gate. (H) Normalized mean fluorescence intensity (MFI) for p38 and p65 per condition. (I) Phosphorylation array of male (M) and female (F) hDRG protein samples (ANOVA:Sex [*P* = 0.0266], Sex:Site interaction [*P* = 2e-06], n = 4 per group). Related to SFigure 9–11, http://links.lww.com/PAIN/C300. FACS, fluorescence-activated cell sorting; hDRG, human dorsal root ganglia; LPS, lipopolysaccharide.

To further support the relevance of TNFα sexual dimorphism in the peripheral nervous system, we investigated TNFα release and signalling in the hDRG directly (Fig. 6).

To determine if myeloid immune cells from DRG parenchyma could be a local source of TNFα, we isolated CD11b+ cells from hDRG through FACS (Figs. 6A and B). Following isolation, cells were stimulated with LPS (10 ng/mL) or vehicle (RPMI media) with or without the intracellular protein transport inhibitor Brefeldin A for 16 hours. In myeloid cells without Brefeldin A, we found a robust increase in levels of TNFα following LPS stimulation (SFig. 10, http://links.lww.com/PAIN/C300, n = 5). The statistical power to test sexual dimorphism (assuming a 2-fold effect size, alpha = 0.1 and n = 2-3) is 0.12 to 0.14 with a 2-sided *t* test (“pwr” package, in R). Given this limited power, we are unable to robustly assess male/female differences based on the variability in the current data.

We complemented this data with myeloid cells co-stimulated with Brefeldin A followed by fixation, permeabilization, and staining for intracellular TNFα production with a TNFα antibody (Figs. 6C and D). Following LPS stimulation, there was a higher percentage of cells with TNFα staining (18.7%) when compared with vehicle-treated cells (0.36%) (Figs. 6C and D). Together, these findings suggest that hDRG myeloid cells can serve as a local source of TNFα production.

### 3.4. Phosphorylation in the human dorsal root ganglia

In parallel, we examined if TNFα stimulation could drive increases in the phosphorylation of transcription factors p38 and p65. CD11b+ cells were stimulated with TNFα (10 ng/mL) or its vehicle (1X PBS) for 30 minutes (Fig. 6E). Cells were then collected, fixed, permeabilized, and stained with p38 and p65 antibodies. We observed a higher percentage of CD11b+ cells stained for p38 and p65 upon TNFα stimulation when compared with vehicle-treated cells (Figs. 6F and G). TNFα-treated cells also exhibited a higher median fluorescence intensity signal of both p38 and p65 antibodies (Fig. 6H), together suggesting that TNFα can drive the phosphorylation of p38 and p65 in the DRG.

Finally, we asked whether phosphorylation in general may be sexually dimorphic (indirectly of TNFα stimulation) in hDRG. We assessed the phosphorylation status of kinases involved in prominent cellular signalling pathways (eg, STAT, EGF, ERK1/2) in matched protein samples from our proteomic experiment (4M + 4F) to query sex-specific phosphorylation differences in our pain-free donors (Fig. 6I). We see significant differences in Sex (*P* = 0.0266), and the Sex:Site interaction (*P* = 2e-06), but we do not see differences for individual phosphorylation sites (Tukey honestly significant difference [Tukey HSD], adjusted *P* ∼ 1 for all sites).

## 4. Discussion

This study explores sexual dimorphism in a proteomics dataset of the hDRG. It complements previous ATAC-seq and RNA-seq work, and with deep coverage spanning over 12,500 gene groups (and ∼9000 unique proteins) per tissue, it provides the most comprehensive proteomic data to date for the hDRG. Despite our small sample size, we report distinct sex differences with a particular focus on TNFα signalling, highlighting that these differences are seen consistently across cohorts and omics technologies. We have paired this to in vitro work on freshly isolated hDRG immune cells, confirming the functional relevance of this pathway in the hDRG. Collectively, this indicates a broad sexual dimorphism in the hDRG, with likely functional and clinical implications.

In addition to providing the first human-centric DIA-MS proteomics reference of the hDRG, we also improve the coverage over preclinical DRG proteomes in terms of ion channel and membrane protein detection.^6^ In this study, we highlight membrane proteins relevant to pain- and neuroimmune conditions, even detecting large membrane proteins like PIEZO1.

We validate this dataset across the nerve root and ganglia, highlighting differences in ion channel expression and myelin-related proteins by tissue. In the nerve root, we see an enrichment of terms related to myelination through “myelination,” “axon ensheathment,” and “gliogenesis,” among others, while also observing an enrichment in ion regulation, including “potassium ion homeostasis,” “intracellular sodium ion homeostasis,” and “sodium ion transmembrane transport.” Fewer GO terms are enriched in the ganglia, these are related to “tRNA aminoacylation” and “amino acid activation” highlighting the role of cell bodies in translation.

While both the nerve and ganglia contain primary afferent components (soma vs axons), mRNAs can be transported from the soma for localized translation in the axons.^47^ These tissues also contain different proportions of non-neuronal cell types, with satellite glial cells wrapping around soma and Schwann cells (both myelinating and nonmyelinating) playing essential roles in axonal integrity. As a result, peripheral nerves and ganglia are uniquely affected in conditions such as diabetic and chemotherapy-induced peripheral neuropathies, and knowledge of their protein setup can lay a foundation for future research.

When probing sexual dimorphism in the ganglia and nerve root, we see a shared pattern of “Oxidative Phosphorylation” enrichment in female donors, as well as differences in “TNFα” and “IFNα” signalling. Notably, we see TNFα signalling enriched in male ganglia across datasets, spanning from different accessible gene regions (through ATAC-seq),^24^ to different transcriptional profiles in human participants.^44^ By detecting this sexual dimorphism across omics types, as well as across samples from organ donors and human participants, we gain confidence in this being a true biological signal. Paired with this, there is strong evidence from clinical trials that TNFα inhibitors affect men and women differently,^13^ suggesting a meaningful molecular signature.

In mice, TNFα signalling in the DRG was recently shown to be sexually dimorphic in an experimental autoimmune encephalomyelitis model.^38^ In humans, this pathway is frequently studied in circulating immune cells, and TNFα signalling here can be regulated by testosterone levels.^36^ Using FACS/flow cytometry on freshly isolated hDRG, we first examine the functional relevance of this pathway in the hDRG specifically: When stimulated, CD11b+ cells from hDRG release TNFα, and TNFα stimulation can alter phosphorylation levels.

Using a targeted phosphorylation approach, we then investigate sexual dimorphism at the level of posttranslational modifications. In this study, we see an interaction of sex and phosphorylation site in the hDRG. Together this provides an initial avenue for follow-up in humans, as previous work in mice by Maguire et al.^38^ report phosphorylation differences in the context of the TNFα signalling pathway dimorphism.

## 5. Limitations

This dataset presents the highest proteome coverage of the hDRG to date and overcomes many of the known difficulties in detecting membrane proteins, but is not complete. We observe ∼20% of ion channels with detectable transcripts in bulk RNA-seq data, and we detect less than 10% of the corresponding GPCRs detected in a comparable bulk RNA-seq dataset.^44^ Prior studies highlight functional expression of membrane proteins such as ASICs, opioid receptors (OPRs), and PIEZO2 in DRG, which are missing in this study. Thus, the lack of detection is likely technical (given their low abundance), as opposed to biological. Increases in sample sizes, paired with further improvements in sensitivity and sample preparation protocols, will facilitate the detection of very low abundant membrane proteins in complex protein mixtures in future work.^43,59^

Proximal and distal nerve regions exhibit prominent differences and are uniquely affected by length-dependent pathologies (eg, diabetic peripheral neuropathy).^22^ Thus, our nerve data likely do not represent the proteomes of distal nerves or terminal endings. As technology develops, added information on peripheral endings in the skin and viscera will also be beneficial, as current proteomics of human skin does not include common neuronal markers.^21^

## 6. Future directions

The full implications of the sexual dimorphism reported here remain unclear: A previous study in healthy trans men shows that testosterone (as hormone replacement therapy, HRT) can shift immune function in plasma samples in line with the dimorphism of the hDRG we report in this study.^36^ Within 12 months of starting HRT, these men show increased TNF signalling through NFκB, paired with a decrease in the IFNα response, highlighting adaptations to immune function in response to increasing testosterone levels.

Beyond TNFα signalling, varying levels of testosterone in cis men correlate to the immune response after vaccination,^25^ and HRT in postmenopausal women can elicit immune changes as early as 1 month.^34^ Hormone differences are highly relevant in autoimmune conditions as well: While HRT in menopausal women may increase the risk of late-onset RA, hormonal oral contraceptives have been shown to reduce the risk of RA in women.^28^

Given the variation across humans and the adaptive nature of the immune system, the differences reported likely exist on a spectrum that can change with medication and age. In the hDRG specifically, the functional implications require more research. We show that TNFα is released from myeloid cells from DRG parenchyma and that TNFα stimulation of hDRG immune cells can result in protein phosphorylation, but a direct causation to sexually dimorphic PTMs—and any downstream functional changes—is still needed.

Moving forward, differing immune signatures in their local environment should be accounted for in future studies, especially when considering developmental markers such as puberty and menopause.

This comprehensive, human-centric proteomic dataset complements previously published ATAC-seq and RNA-seq data, providing a quantitative reference proteome of the hDRG. Data are searchable at https://sensoryomics.com/.

Using tissue from both human participants and donors, we show evidence for sexual dimorphism in TNFα signalling spanning the epigenetic to proteomic signature. Taken together, these results underscore a relevant sexual dimorphism in the hDRG, with significant implications for sensory and pain-related translation.

## Conflict of interest statement

The authors declare no conflicts of interest related to this work. D.G.V. and M.S. have an ongoing scientific collaboration with Bruker (Center of Excellence for Metaproteomics); however, this collaboration did not influence the content of the manuscript.

## Supplemental digital content

Supplemental digital content associated with this article can be found online at http://links.lww.com/PAIN/C301 and http://links.lww.com/PAIN/C300.

## Supplemental video content

A video abstract associated with this article can be found on the PAIN Web site.

## Supplementary Material

SUPPLEMENTARY MATERIAL
