# Supplementary material for: Multi-omic integration with human dorsal root ganglia proteomics highlights TNFα signalling as a relevant sexually dimorphic pathway
Source: Pain. 2025 May 20;166(10):2386–402. doi: 10.1097/j.pain.0000000000003656 (PMC12353054; doi:10.1097/j.pain.0000000000003656)
Supplement: SUPPLEMENTARY MATERIAL [file jop-166-2386-s002.pdf]

Supplemental Tables

Table	Contents
Supplemental Table 1	DEP-tissue (ganglia vs nerve)
Supplemental Table 2	GO-tissue, up in ganglia
Supplemental Table 3	GO-tissue, up in nerve
Supplemental Table 4	DEP-sex, ganglia (MvF)
Supplemental Table 5	GSEA-sex, ganglia (padj < 0.05)
Supplemental Table 6	DEP-sex, nerve (MvF)
Supplemental Table 7	GSEA-sex, nerve (padj < 0.05)
Supplemental Table 8	GSEA-MSFA, factor3 (padj < 0.05)
Supplemental Table 9	Phosphorylation Array, source data
Supplemental Table 10	Antibody details for FACS
Supplemental Table 11	Proteomic expression matrix

DEP; differentially expressed proteins

Supplemental Figures

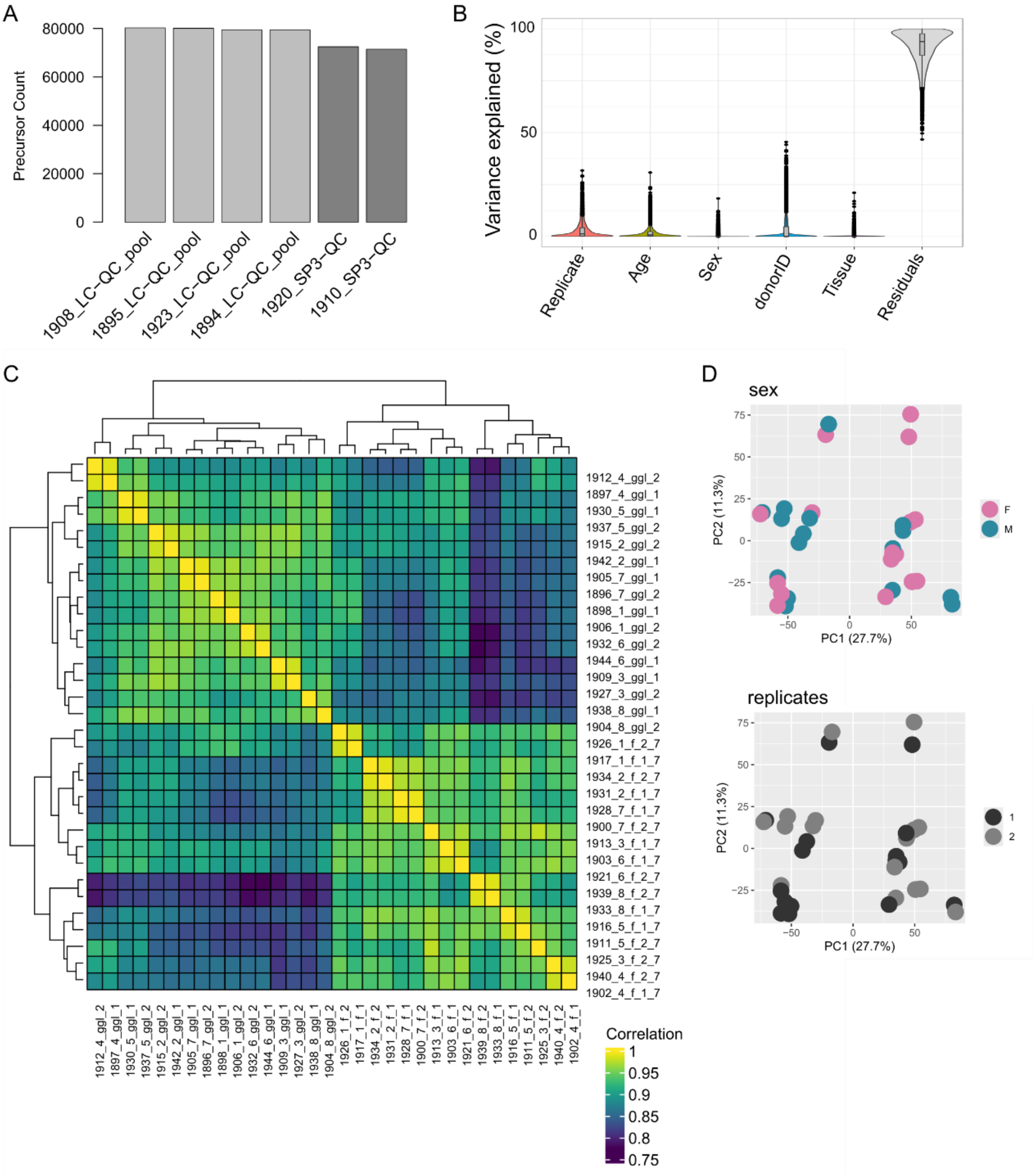

SFigure 1. Quality control of the hDRG DIA-PASEF dataset. A. Precursors (q < 0.01) across reference quality control samples. B. Variance partitioning across all biological samples. C. Correlation across all biological samples (replicates as _1 or _2). D. PCA by sex and replicate for unmerged technical replicate samples.

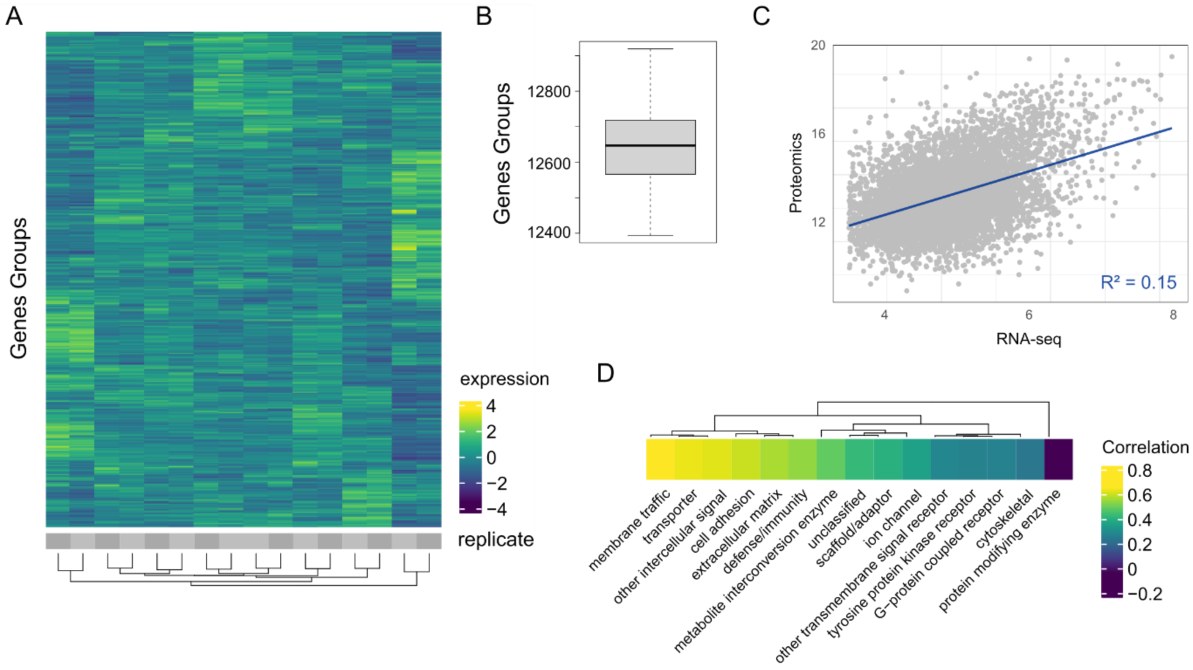

SFigure 2. Quality control for the ganglia. A. Gene Group expression across replicates. B. Gene Group counts per sample. C. Correlation with hDRG RNA-seq data (average protein expression and average quantile-normalized transcripts per million (qnTPM)). D. Correlation with hDRG RNA-seq data separated by receptor group (min 15 proteins per group).

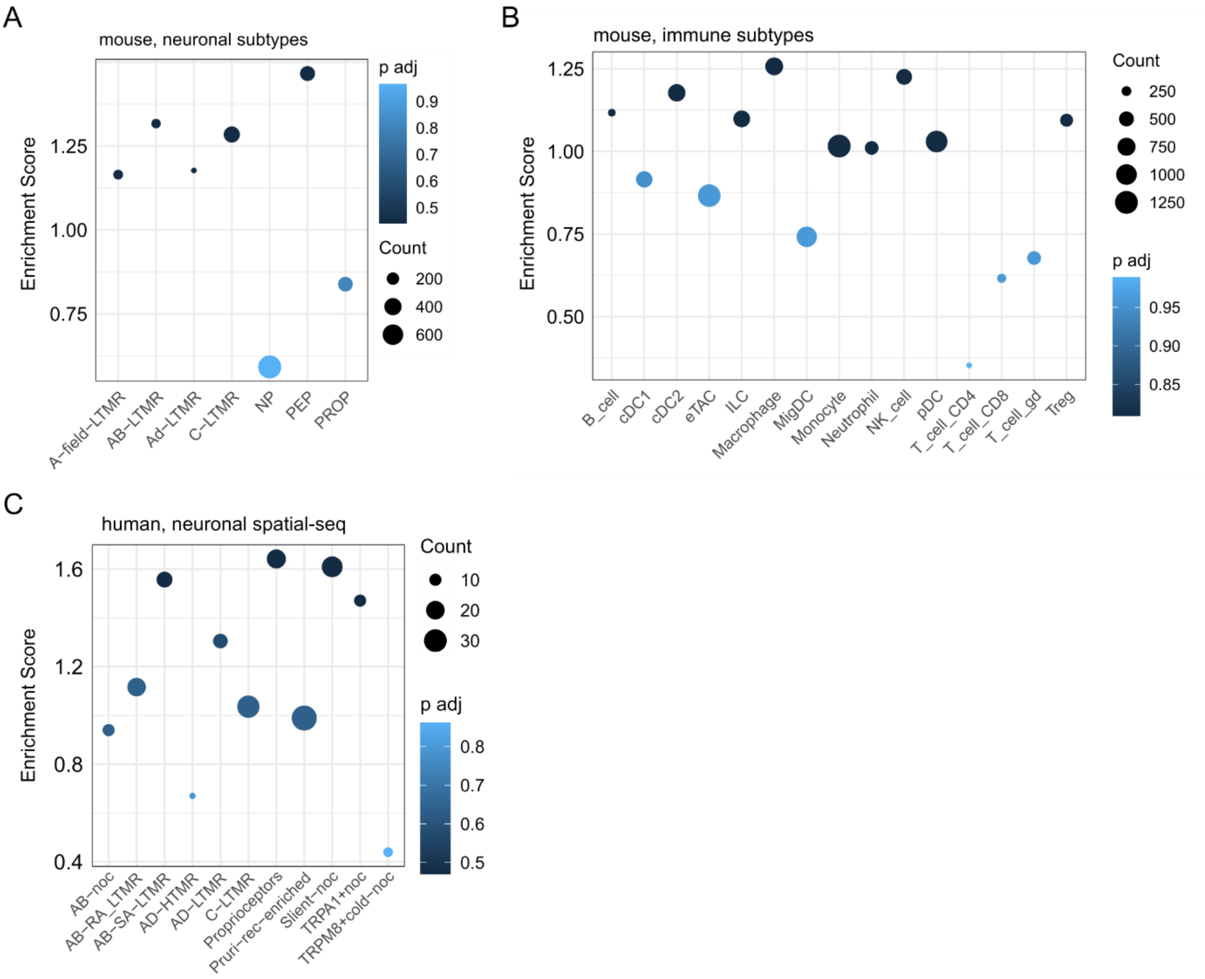

SFigure 3. Gene set enrichment analysis (GSEA) for: A. mouse, neuronal. B. mouse, immune cells. C. human, neuronal gene sets against the hDRG proteome.

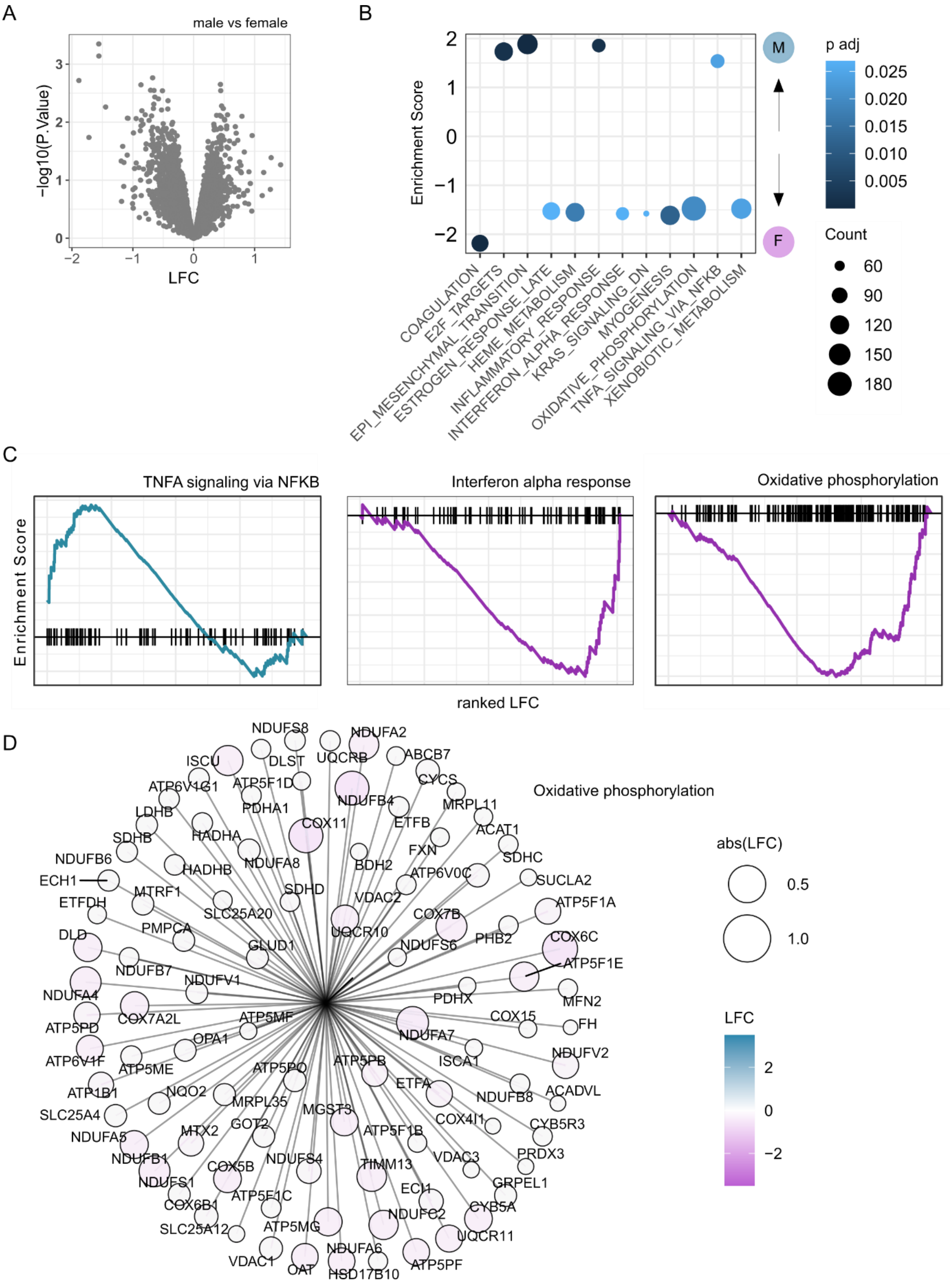

SFigure 4. Sexual dimorphism in the hDRG. A. Volcano plot of DEPs. B. GSEA against hallmark pathways, males = positive. C. Representative gene rankings from B for pathways of interest. D. LFC for proteins in the `Oxidative Phosphorylation` pathway. LFC = log fold change.

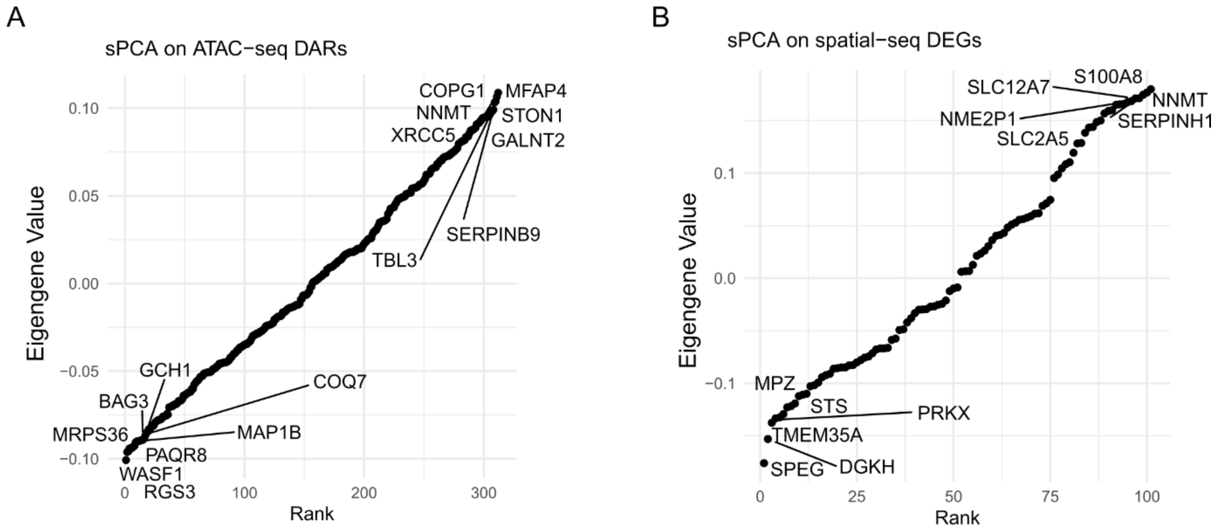

SFigure 5. sPCA eigengenes from A. ATAC-seq DARs and B. spatial-seq DEGs, with tails labelled.

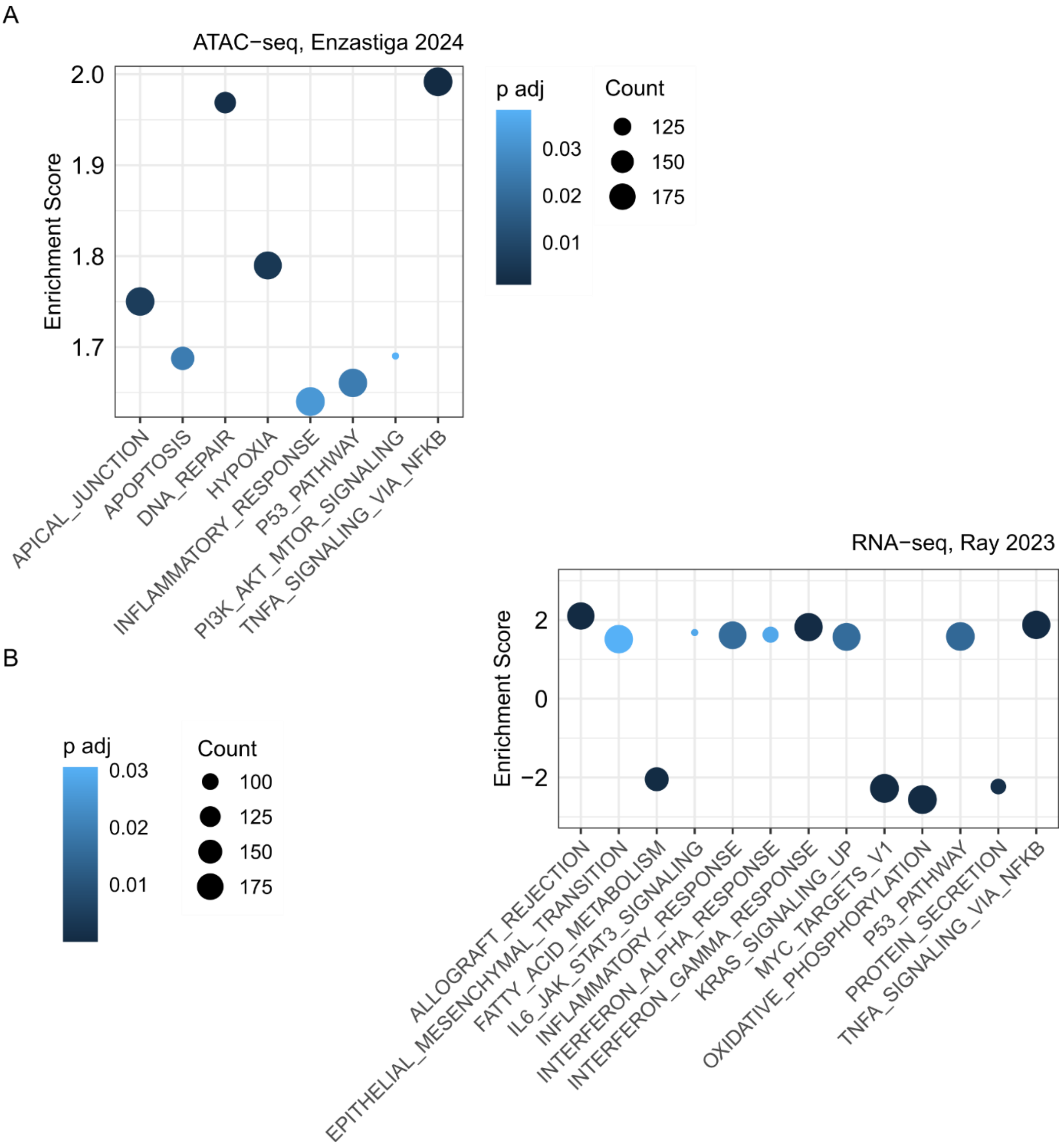

SFigure 6. GSEA against previously published ATAC-seq and RNA-seq datasets [21,37]. A. ATAC-seq on donor samples, LFC for pseudobulk ATAC-seq spatial data. B. RNA-seq on participant samples.

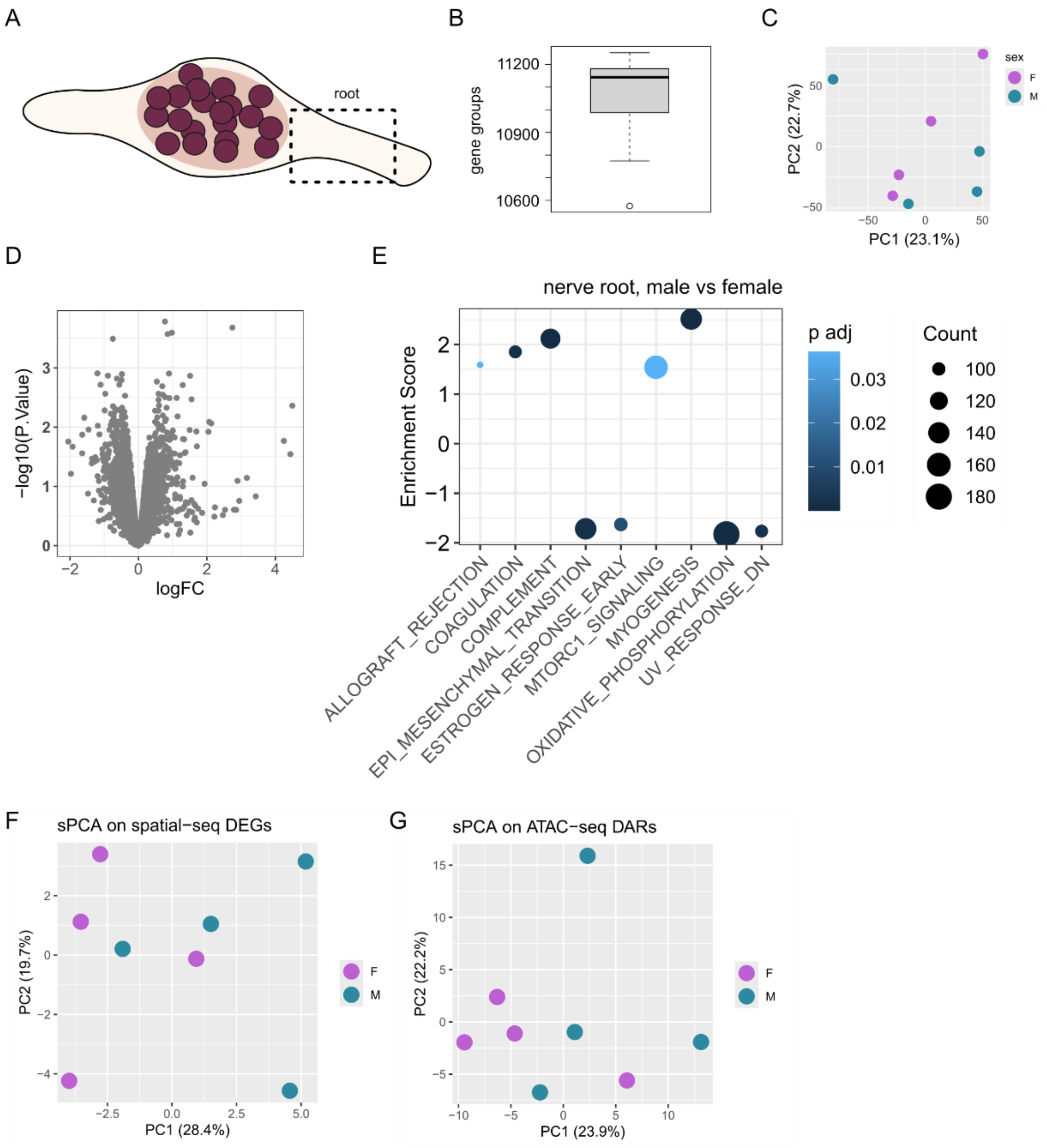

SFigure 7. Proteome of the nerve root. A. Schematic. B. Gene Groups per sample. C. PCA by sex. D. Volcano plot, differential expression testing with limma, male = positive. E. GSEA against Hallmark pathways, male enrichment = positive. F-G. supervised PCA (sPCA) on differentially expressed genes (DEGs, F) and regions (DARs, G) from previously published reports on hDRG sexual dimorphism.

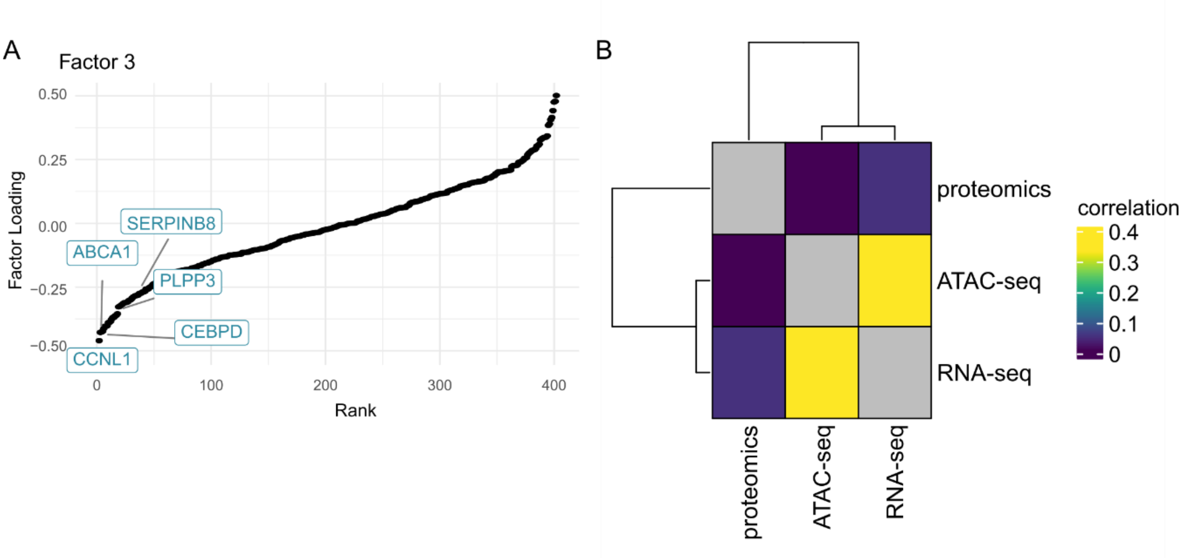

SFigure 8. Multi-study factor analysis (MSFA) across omics datasets. A. Ranked factor loadings from Factor 3, with terms associated with TNFα signalling highlighted in the tail. B. Correlation between LFC for matched accessible regions, genes, and proteins, LFC for pseudobulk ATAC-seq spatial data.

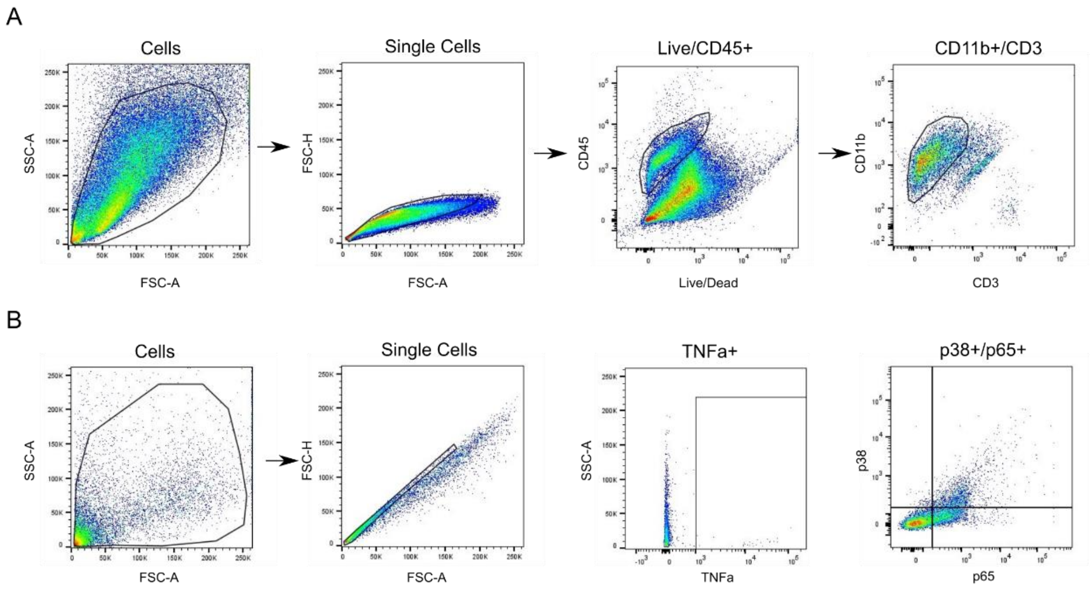

SFigure 9. Gating strategies used in flow cytometry experiments. A. Depiction of the gating strategy used to isolate human dorsal root ganglia myeloid cells using fluorescently activated cell sorting. B. Depiction of the gating strategy used to measure TNFα production and phosphorylation of p38 and p65.

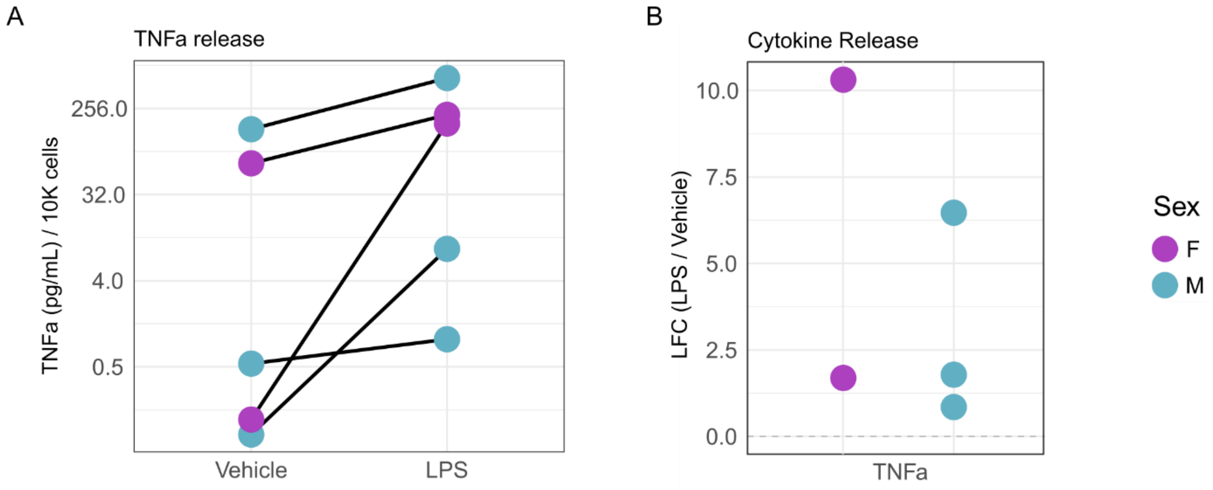

SFigure 10. TNFα is released from stimulated myeloid cells from DRG parenchyma. A. TNFα (pg/mL), normalized per 10000 cells plated from Vehicle-treated or LPS-stimulated cultures. B. Log2 fold change (LFC) of TNFα amount from LPS / Vehicle cultures (n = 2F + 3M).

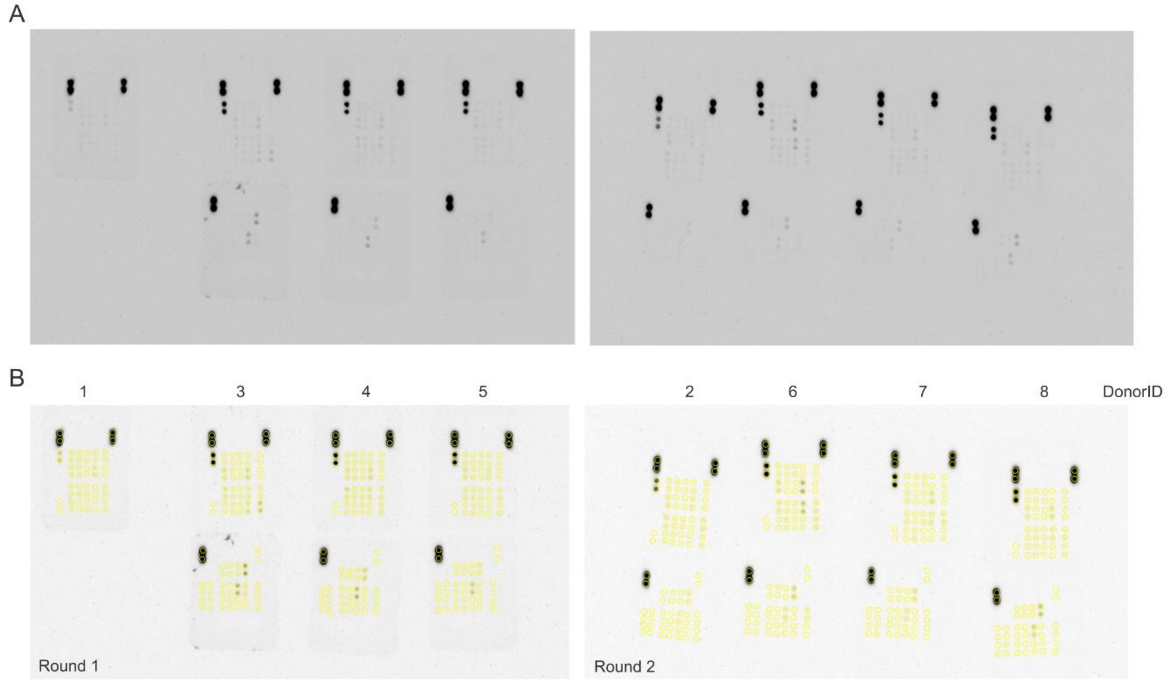

SFigure 11. Phosphorylation Array membranes. A. 240 second exposure for round 1 (left) and 2 (right), 2M/2F per round (age-matched, except for Donor 1, where only membrane "A" was processed). B. Selected ROIs (in yellow) for each phosphorylation site, donor numbers match "MS-" donors in Table 1. Contrast enhancement for visualization only. Corresponding source data in Supplemental Table 9.
